# Tissue-Specific Decellularization Methods: Rationale and Strategies to Achieve Regenerative Compounds

**DOI:** 10.3390/ijms21155447

**Published:** 2020-07-30

**Authors:** Unai Mendibil, Raquel Ruiz-Hernandez, Sugoi Retegi-Carrion, Nerea Garcia-Urquia, Beatriz Olalde-Graells, Ander Abarrategi

**Affiliations:** 1 Center for Cooperative Research in Biomaterials (CIC biomaGUNE), Basque Research and Technology Alliance (BRTA), 20014 Donostia-San Sebastian, Spain; umendibil@cicbiomagune.es (U.M.); rruiz@cicbiomagune.es (R.R.-H.); sretegi@cicbiomagune.es (S.R.-C.); 2 TECNALIA, Basque Research and Technology Alliance (BRTA), 20009 Donostia-San Sebastian, Spain; nerea.garcia@tecnalia.com (N.G.-U.); beatriz.olalde@tecnalia.com (B.O.-G.); 3 Ikerbasque, Basque Foundation for Science, 48013 Bilbao, Spain

**Keywords:** extracellular matrix, decellularization, regenerative medicine

## Abstract

The extracellular matrix (ECM) is a complex network with multiple functions, including specific functions during tissue regeneration. Precisely, the properties of the ECM have been thoroughly used in tissue engineering and regenerative medicine research, aiming to restore the function of damaged or dysfunctional tissues. Tissue decellularization is gaining momentum as a technique to obtain potentially implantable decellularized extracellular matrix (dECM) with well-preserved key components. Interestingly, the tissue-specific dECM is becoming a feasible option to carry out regenerative medicine research, with multiple advantages compared to other approaches. This review provides an overview of the most common methods used to obtain the dECM and summarizes the strategies adopted to decellularize specific tissues, aiming to provide a helpful guide for future research development.

## 1. Introduction

**Figure 1:**
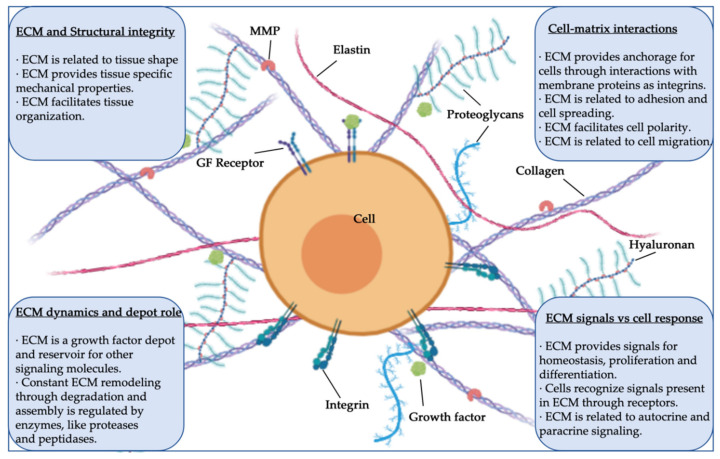
Structure, components, and functions of extracellular matrix (ECM) (MMP, matrix metalloprotease; GF, growth factor).

In many adult animal tissues, the main component in terms of volume is not the cells, but the cell-secreted three-dimensional (3D) structure known as the extracellular matrix (ECM). Structural and specialized proteins and proteoglycans are some of the ECM’s macromolecular components, and they interact in this network with multiple key roles and functions. During homeostasis, the ECM provides structural integrity and mechanical support for tissues and organ architecture. In parallel, the ECM is the reservoir and the place for the active exchange of ions, nutrients, waters, metabolites, and signals [1]. In this way, the ECM serves as the environment in which tissue-resident cells attach, communicate, and interact, thereby regulating cell dynamics and behavior, and contributing to the maintenance of tissue-specific cell phenotypes and functions (Figure 1). Notably, the ECM provides a niche for tissue-resident stem cells and drives their fate decisions, a property particularly relevant during homeostasis and tissue repair–regeneration processes.

The properties of the ECM have been thoroughly used in tissue engineering and regenerative medicine research, aiming to restore the function of damaged or dysfunctional tissues [2]. In this context, applications of ECM-derived components are multiple, from in vitro stem cell basic research to clinical settings. For example, ECM-derived components have been used as surface coatings for cell adhesion purposes; gel matrices for establishing organoid cultures; 3D environments for cell seeding and growth factor delivery approaches; and as biocompatible and biomimetic implantable allograft or xenograft scaffolds with in vivo tissue regeneration properties. Often, strategies are based on specific ECM components, such as collagens, fibrin, hyaluronic acid, or even cell-culture-derived ECM. Using these materials, multiple fabrication techniques have been implemented to generate successful ECM-derived biomimetic structures such as hydrogels, or even more sophisticated engineering approaches such as micropatterned surfaces, electrospinning, 3D printing, and bioprinting-designed scaffolds, among others [3].

**Figure 2:**
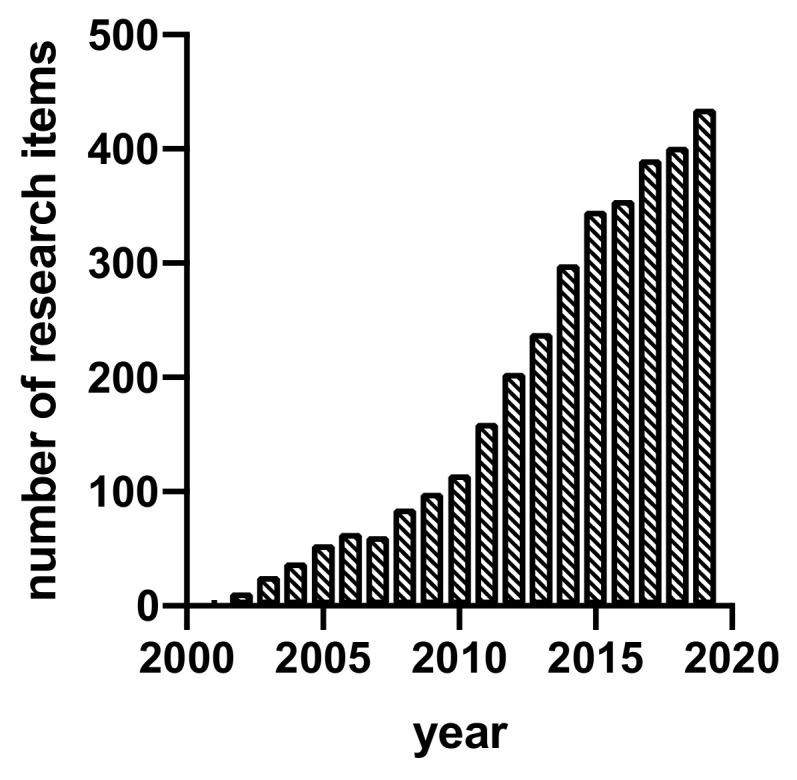
Research items per year with the words “decellularization” or “decellularized” in the title (Source: https://scholar.google.es “allintitle: decellularized OR decellularization”).

Interestingly, tissue decellularization is becoming a common technique to obtain decellularized extracellular matrix (dECM) [4] and it is a research field gaining momentum in recent years (Figure 2). The rationale for decellularization is related to the adverse response that cell waste may induce when tissue-derived material is used for implantation procedures, including immune reaction and inflammation, leading to implant rejection. Therefore, dECM is usually obtained by chemical, enzymatic, and/or physical decellularization methods, developed to eliminate the cells and their waste, mainly DNA [5]. These procedures yield decellularized materials formed by the multiple ECM components, which are maintained similar to the original tissue in composition, even in architecture, if required. dECM-related advantages are often associated with better performance and applicability as implants for tissue repair, and also with better mechanical/biochemical properties for the intended use. Initially, research in this field was focused precisely on developing proper decellularization methods and techniques, while more recently, the field is moving to implement approaches related to bioengineering and which tackle applied research aims.

Notably, regenerative medicine research can now take advantage of approaches based on the use of the targeted tissue-specific dECM [6,7]. The ECM is different from one tissue to another, and therefore, decellularization methods and techniques to obtain dECM have been extensively studied and tissue-specifically improved, aiming to preserve the ECM molecules and structures relevant for the intended use. In this sense, research has been mainly based on empirical testing of tissue-specific decellularization methods intended to achieve specific aims or applications.

In this review, we provide an overview of the methods used to obtain the dECM, and we summarize the most common strategies adopted to decellularize specific tissues, aiming to provide a helpful guide for future research development.

## 2. Organ Decellularization and Tissue Decellularization Approaches for Biomedical Applications

Whole tissue or intact tissue pieces or sections are a common starting point for decellularization, especially when the final purpose is whole organ decellularization for bioengineering purposes. In these cases, the resulting dECM is a tissue scaffold generally created to keep its structure as intact as possible. Note that dECM tissue scaffold decellularization processes tend to be long, due to the need to be sure about all the reagents reaching the target cells in order to achieve complete decellularization [5]. The bigger the tissue pieces, the longer it takes to make sure that they are completely decellularized. Moreover, the longer the period of chemicals and enzymatic reactions, the higher the chance of damaging the ECM components.

In tissue pieces, it is easy to assess by histology the decellularization and integrity of the remaining ECM. However, even if there are no histologically visible nuclei in the tissue, it is still important to quantify the DNA content by molecular techniques, with the safe limit of DNA content in a decellularized tissue established as below 50 pg DNA per milligram of dry tissue. Regarding macromolecules, they need to be assessed both in quantity and quality by histology, spectrophotometry, and other techniques, while the other ECM components as growth factors may need to be assessed and quantified as well [8]. In some cases, tissue-specific tests are also required to characterize those properties key to the intended biomedical application. For example, a mechanical stress test is required to assess the mechanical/elastic properties after the decellularization of tendons, muscles, and cartilage tissues [9].

Keeping the ECM as intact as possible can be a problem when dECM tissue scaffolds are meant to be used for cell colonization purposes [10]. The ECM is grown by and around the cells, providing physical support, and therefore keeping intact the intricate ECM net, which may impede proper cell seeding of decellularized material. Moreover, potential implantation may be physically limited by the dECM structure and form. Therefore, the decellularization strategy is often designed to degrade some of the ECM components, aiming to maximize the further cell seeding strategy [11].

Tissue decellularization is achieved using as starting material tissues treated with mechanical or chemical methods for tissue grinding, pulverization, or homogenization before decellularization. This approach is gaining relevance, especially in strategies aiming to use the dECM in postprocessing fabrication approaches (hydrogels, 3D printing, electrospinning, and similar). The outcome of decellularization in these cases is dECM powder, an intermediate product mainly used to artificially generate further ECM coatings or 3D structures [12]. Note that tissue powder processing yields dECM powder with multiple components, but does not keep the tissue architecture and affects the structure of the ECM macromolecules. This is because the aim of approaches using this processing method is not to keep the structural proteins untouched, but rather to use the properties of the relevant ECM components to improve biocompatibility, adhesion, differentiation, and/or other properties or purposes.

The applications of decellularized materials and matrices in regenerative medicine context are multiple, including clinically used implantable materials, and continue to expand. For example, whole decellularized pieces are mainly used as scaffolds for transplantation purposes; dECM processed to form sheets and/or patches is useful in soft tissue and cardiac repair; Powder of demineralized bone matrix can be resuspended and be used to fill and heal bone defects; dECM-derived hydrogels are useful as injectable materials with regenerative properties; Hydrogels can be processed too, to generate inks and bioinks useful in 3D printing and electrospun-based strategies; dECM-derived scaffolds can be used as cell carriers for in vitro modeling or in vivo regenerative purposes [2,13,14,15,16,17,18,19].

**Figure 3:**
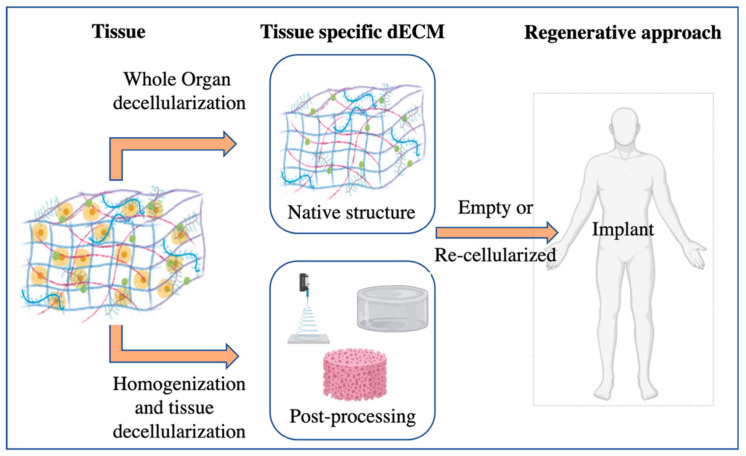
Schematic of organ decellularization and tissue decellularization approaches.

In any case, the decellularization procedure selected and the further characterization needs depend on the final aim and approach (Figure 3). Whole organ, tissue pieces, or powdered ECM are the most common starting materials and multiple possible decellularization methods can be applied, all of them with advantages and disadvantages to be taken into account in light of the specific aim and context [18].

## 3. ECM Decellularization and Sterilization Methods

Most protocols describe combinatorial and sequential use of different physical, chemical, and enzymatic techniques in order to achieve tissue-specific decellularization. In general, chemical and enzymatic techniques are mainly responsible for successful decellularization in most protocols. Physical techniques are generally used to complement chemical and enzymatic techniques and therefore increase the decellularization effects (Table 1). Physical techniques can produce damage in the matrix, while chemical techniques can produce reactions that change the chemical composition of the ECM [20,21,22]. For this reason, setting up the decellularization protocol is of paramount importance in each specific approach.

### 3.1. Chemical and Enzymatic Methods for Decellularization

Detergents are chemical agents used to solubilize cell membranes and to dissociate their inner structure. Among them, Triton X-100 is the most commonly used detergent in decellularization processes. It targets the lipid–lipid and lipid–protein interactions, but it leaves the protein–protein interaction intact [9,23]. It is a very useful agent in those tissues where the key matrix components are primarily proteins. It is an effective detergent to eliminate cells from many tissues, but it is generally avoided in tissues with glycosaminoglycans (GAGs) as a key component in their matrix.

Side by side with the Triton X-100, sodium dodecyl sulfate (SDS) is the other most commonly used detergent in decellularization procedures. SDS solubilizes both the external and nuclear membranes, but also tends to denature proteins and may alter the native structure of the matrix [24,25]. For these reasons, short time SDS treatment is the most common, aiming to minimize the possible damage to proteins and the overall matrix structure [26]. Nevertheless, it is very efficient in removing nuclear and cytoplasmic waste.

Other detergents are useful for specific tissues and applications in a decellularization context. CHAPS reagent has properties of both ionic and nonionic detergents, and therefore, it targets lipid–lipid and lipid–protein interactions, while also solubilizes membranes [27]. CHAPS is not good as a permeating agent, and is therefore mainly used to decellularize thin tissues, for which it is more effective. Triton X-200 is another detergent, less used than its X-100 counterpart because it is more prone to alter the ECM’s structure, but it is highly effective for tissues such as neural tissue [28].

Enzymes are also used in most decellularization protocols, mainly to eliminate cell waste and other undesirable components of the ECM. However enzymatic treatments can often lead to additional problems related to enzyme removal [20], a problem usually tackled with further steps using nonenzymatic agents or detergent treatment. Among such enzymes, trypsin is the most commonly used in decellularization procedures [29]. Trypsin targets the C-side bonds in arginine and lysine amino acids and is mostly used combined with EDTA, a chemical agent able to break cell–matrix interactions. Of note, the prolonged exposure to trypsin–EDTA treatment can significantly alter the structure of the matrix, destroy laminin, and remove GAGs, resulting in severe mechanical weakness of the tissue [30]. Pepsin is another useful enzyme in decellularization processes. It is a highly aggressive protease commonly found in the stomach and, like trypsin, it targets the bounds between peptides. Thus, it may cause damage to the ECM if used under long exposition times [31].

Hypotonic and hypertonic solutions [32] use osmotic properties to make the cells explode. The osmotic shock kills the cells, but it does not remove the cell waste that it releases to the matrix, which should be taken into account in the design of a decellularization procedure. Moreover, the treatment of the DNA waste is of paramount relevance in all decellularization processes, due to the tendency of the nuclear material to remain stuck to ECM proteins. In this sense, endonucleases and exonucleases are other kinds of enzymes that are of great use to eliminate the waste of nuclear components [8,30].

Finally, chemical acid–base [33] and TBP [32] treatments are rarely used, because they are very aggressive toward the proteins of the matrix. Specifically, acid–base solutions damage collagen and TBP disrupts protein–protein bonds.

### 3.2. Physical Methods for Decellularization

As previously mentioned, physical techniques are not enough to decellularize the tissue, but they can help in combination with chemical and enzymatic processes. For example, when big tissue pieces or whole organs are the target of decellularization, perfusion is recommended in order to better reach all tissue areas [21,34].

The most commonly used physical technique is snap freezing, or freeze–thawing, as the first step of a decellularization process. By freezing tissue, intracellular ice crystals are formed, thereby disrupting cellular membranes and causing cell lysis. Thus, freezing is a common and effective method for cell lysis and it eases further uniform decellularization. Protocols using this approach have to carefully control the rate of temperature change to control the size of the formed ice crystals, therefore preventing excessive damage to the ECM [35,36,37,38].

Cells can be lysed by applying direct pressure to tissue, but this method is only effective for tissues or organs that are not characterized by a densely organized ECM (e.g., liver and lung). Mechanical force has also been used to delaminate layers of tissue from organs that are characterized by natural planes of dissection, such as the small intestine and the urinary bladder. These methods are effective and cause minimal disruption to the three-dimensional architecture of the ECM within these tissues [39].

Mechanical agitation and sonication are useful in combination with a chemical treatment to assist in cell lysis and the removal of cellular debris [40,41]. Mechanical agitation can be applied by using a magnetic stir plate, an orbital shaker, or a low-profile roller. There are no studies to determine the optimal magnitude or frequency of sonication for the disruption of cells. However, the standard ultrasonic cleaner appears to be as effective at removing cellular material as the movement of an orbital shaker. In all of these procedures, the optimal speed, the volume of reagent, and the length of mechanical agitation are dependent on the composition, volume, and density of the tissue.

Vacuum-assisted decellularization (VAD) cannot decellularize, but it is highly effective in enabling chemical agents to reach the whole tissue [42]. Hydrostatic pressure, on the other hand, is an effective decellularization method, but it is usually combined with enzymes such as DNases to achieve complete decellularization [43].

### 3.3. Sterilization Methods

The dECM is commonly used in in vivo implantable approaches. Therefore, in order to prevent the transmission of pathogens, the dECM needs to be sterilized to eliminate any microorganisms and to prevent infections [60]. There are some useful physical and chemical dECM sterilization techniques, although their suitability depends on multiple factors, which may need to be considered in each specific approach; for example, humidity, time of exposure, temperature, and the nature/load of the bioburden are some of the factors to be taken into account.

Among the physical methods, two groups are differentiated—those using heat and those using radiation. Heat techniques have very limited use in a dECM context, as dECM products are usually thermosensitive and high temperatures may denaturalize important proteins, as well as disrupt the structure [61,62]. Irradiation effectively eliminates microorganisms, and UV-irradiation is a common sterilization method used in research settings, mainly due to easy accessibility in terms of research labs and cell culture facilities [63]. On the other hand, gamma-irradiation is the preferred sterilization method for many pharmaceutical and clinical products, due to its high penetration, good assurance of sterilization, and feasible temperature during the sterilization process. Conversely, radiation may affect structural proteins such as collagen, reducing the strength of the treated material [64,65,66]. Therefore, gamma-irradiation is often selected as the sterilization method for dECM products, but the irradiation dosage has to be optimized for each specific case, and the properties must be assessed after treatment.

Liquid chemical wash can be used as a sterilization method. Alcohol-based sterilization is common in laboratories, as it is cheap and easy to access. This method is more effective at killing microbes in aqueous solutions, but may also affect the protein structure in dECM pieces. Phenols act by disrupting membranes, precipitating proteins, and inactivating enzymes. They are bactericidal, fungicidal, and mycobactericidal, but are ineffective against spores and most viruses. Aldehydes are alkylating agents that damage nucleic acids and kill all microorganisms, including spores. In contrast to alcohols, which are volatile, phenols and aldehydes are generally toxic, corrosive, and/or irritating. Liquid chemicals must be removed after the sterilization process due to their potential toxicity in further in vivo uses [60].

Chemical methods are also useful to sterilize dECM materials, and are an alternative to physical methods. Ozone and hydrogen peroxide are traditional sterilization gases, but ethylene oxide is more commonly used in a dECM context, as the ultrastructure and the mechanical properties of the dECM are usually not altered under such treatment [64,67]. After the sterilization process, it is important to ensure the elimination of residual sterilizing agents and other possible volatile residues.

## 4. Decellularization Methods by Tissue

In order to choose a decellularization method, the tissue itself and the strategy to be followed have to be taken into consideration. Since each tissue has a different structure and composition, the decellularization methods have to be specifically selected and empirically tested. Moreover, if the aim is to keep the tissue structure as intact as possible, chemical methods have to be chosen carefully because of the damage that they may cause to the structural macromolecules of the matrix. Therefore, the literature shows trends in the use of specific combinatorial physical and chemical approaches for each specific tissue and application, as follows.

### 4.1. Bone Tissue

Bones are the main components of the skeletal system. They give support to the body, allow movement, and produce red and white cells in their marrow. Bone is a connective tissue and its ECM is formed by different key proteins that confer tensile strength, such as collagen type I, noncollagenous glycoproteins, and proteoglycans. Calcium hydroxyapatite (HA) in the ECM serves to store minerals and provides specific properties to bone tissue, such as resistance and hardness [68], while a series of signaling molecules, such as bone morphogenetic proteins (BMPs), are also part of a bone’s ECM compartment.

The properties of bone’s ECM as regenerative material have been thoroughly described in the literature, and that is why diseased and damaged bones are frequently approached using bone grafts. Bone autografts are the best option in order to avoid rejection, while allografts and xenografts are often used [46,69,70,71,72]. Implantable ceramic scaffolds are also frequent in clinical settings as osteoconductive materials, and some of these products are bovine or porcine bone HA calcareous matrices obtained after heat-treating bones in a muffle furnace to remove all organic compounds, including cells [68,73].

The first use of decalcified bone as bone implantable material was described as early as 1889 by Senn [74], when he used muriatic acid as a decalcification agent, followed by washing and alcohol sterilization before implantation in human bone defects. However, the experimental evidence of the demineralized bone matrix (DBM) as an osteoinductive material was established by Urist in 1965 [52]. At present, osteoinductive activity in the bone matrix is largely related to some of the BMPs present in bone’s ECM, and therefore, it is known that demineralization processing has to be done with the aim to preserve BMPs’ biological activity. In any case, the DBM obtained by different decalcification methods is commercially available and extensively reported in clinical settings as an osteoinductive implantable material suitable for treating bone defects [70,75,76]. Hydrochloric acid (HCl) and EDTA are common decalcifying agents, while chloroform and methanol can be used for lipid extraction. Then, the DBM can be snap frozen, lyophilized, or kept at −20 °C until necessary [71,77].

The decellularized bone matrix (DecBM) is frequently achieved by chemical methods, such as EDTA in combination with trypsin or SDS, along with ammonium hydroxide [46,70]. Alternatively, thermal shock can be used, together with Triton X-100, for effective osseous tissue decellularization [44]. Additionally, high-hydrostatic pressurization, a physical method, has been used with good results regarding bone decellularization [60]. Nucleases and dehydrated alcohol are used as complementary and final steps in order to remove waste nuclear acids and other cellular remains [53,54]. 

Some authors have described specifically the serial decalcification and decellularization steps in their protocols toward the generation of cell-free demineralized implantable materials [71]. In this sense, hydrogels made from the ECM of decalcified and decellularized bone are quite common due to its versatility and osteoconductivity [53,70].

### 4.2. Cartilage Tissue

Cartilage in adult animals is a connective, smooth, and resilient tissue. Hyaline cartilage is avascular, and it is present in the stress points of skeletal tissue, such as bone heads, where it provides flexibility and prevents abrasion and damage [77]. It is also present in the rib cage, nose, larynx, and trachea, while its extracellular matrix is composed of collagens, mainly collagen type II, glycosaminoglycans (GAGs), and laminin [78]. GAGs are closely related to cartilage mechanical properties and help the tissue to cope with sudden external forces [24]. Elastic cartilage is a supportive structure for tissues such as the outer ear and epiglottis, and histologically, it is similar to hyaline cartilage, but with much more elastic fibers [21]. On the other hand, fibrocartilage is the only cartilage with collagen type I in its structure, because it is a mixture of fibrous tissue and cartilaginous tissue, with unique toughness and elasticity properties, and is present in intervertebral discs and menisci, among others. 

Hyaline cartilage is the most commonly targeted cartilage in regenerative treatment—in particular, the one covering the end of bones in articulations. Damage to articular cartilage is usually related to trauma or pathology, and can cause pain, osteoarthritis, or even loss of functionality [78]. Surgical interventions attempt to solve these conditions, but at present, they are largely temporary solutions, pushing research toward searching for new regenerative approaches. The lack of vascularization greatly limits the number of nutrients and oxygen that can reach the inner parts of the tissue. This condition makes cartilage regrowth particularly challenging [79]. Therefore, tackling articular cartilage regeneration is a challenging goal. The literature is extensive in terms of approaches aiming to regenerate articular cartilage tissue, using different kinds of implantable scaffolds [80,81]. In this sense, cartilage dECM derivatives have been used for coating other implantable materials or as a 3D-printed structure for knee and meniscal regeneration, among others.

The basics for articular cartilage decellularization are the same for the different types of cartilages, and in general, decellularization aims to keep in the matrix as many GAGs as possible. Of note, as the different cartilages have different permeability properties, it is paramount to set up proper conditions for optimum decellularization in each case.

Tissue snap freezing or freeze–thawing is a common pretreatment in many decellularization protocols [24,58]. Similarly, pretreatment with hypotonic and hypertonic solutions is also a popular method, used to induce apoptosis by osmotic pressure [11]. These methods do not decellularize the structure by itself, but they help further decellularization methods to work better and to reach the inner parts of the tissue. It has been proved that snap freezing does not affect the matrix component, and it has no significant negative impact on the structure [82]. Conversely, initial tissue homogenization, suggested in some articles, leads to the significant loss of GAGs and structural proteins [83].

Further to physical pretreatments, decellularization has been assayed with enzymatic or chemical detergent methods. The enzyme trypsin–EDTA is the most common decellularization approach reported [83]. Trypsin–EDTA breaks both the proteins that hold the cell in place inside the matrix, as well as the cell membrane proteins. Based on protease activity, the main setback of using this technique is the degradation of the proteins of potential interest in the final dECM. In order to prevent this, the exposure time to trypsin–EDTA needs to be highly controlled, and it is usually limited to 6–24 h [51].

Regarding the detergents used as decellularization reagents, Triton X-100 and SDS are the most commonly used ones. Both cause some extent of damage to the structure of the matrix, but the mechanical integrity of the matrix maintains at an acceptable level. Note that both incur damage to GAG content and integrity, being Triton X-100 the worse in this sense [26,45].

An additional issue to be taken into account regarding cartilage tissue is the need for enzymatic treatment with nuclease activity to prevent waste nucleic acid material from sticking to the matrix [11]. In elastic cartilage, the nuclease time may be necessarily longer to ensure decellularization. In articular cartilage, on the other hand, being a less dense structure, the risk of having DNA material stick to the protein matrix is lower.

The digestion of GAGs using chondroitinase ABC (ChABC) has been reported as a cartilage decellularization method. It is not a commonly used technique; although it facilitates the removal of native chondrocytes, it reduces the mechanical properties of the tissue as well [11].

### 4.3. Adipose Tissue

White adipose tissue is defined as a connective tissue that stores energy in the form of lipids (triglycerides), insulates the body, and provides cushioning and support for subcutaneous tissues and internal organs. It is composed of clusters of fat-storing cells (adipocytes) surrounded by a reticular fiber network and interspersed small blood vessels. The key ECM proteins of adipose tissue are collagen type I, collagen type IV, and laminin. Collagen with laminin provides anchoring sites and barrier functions for adipocytes. Collagen types IV and VII and laminin are major components of the basement membrane [84].

Reconstruction of soft tissue defects is needed after certain tumor resections, external injury, or due to congenital malformations, and presents a major challenge in plastic and reconstructive surgery. At present, the main complications related to adipose tissue reconstruction include capsular contracture, necrosis and donor site morbidity, and immune rejection, and therefore, new clinical approaches are required to improve the success rate. The subcutaneous adipose tissue discarded from surgical operations represents an abundant and easy-to-collect human tissue source, processable by dECM biomaterial [85]. In this sense, allograft and xenograft dECM biomimetic scaffolds have proved to be effective tools for promoting tissue repair and regeneration in numerous preclinical and clinical studies [86,87,88].

The optimal adipose tissue decellularization includes the extraction of lipids (delipidation), followed by the extraction of cells and cell components, thereby maintaining key proteins and the 3D structure. Human and porcine are the most common sources of adipose tissue extraction, and there are two different kinds of initial adipose tissue samples useful for decellularization purposes. Such samples can be solid tissue derived from resection surgery, usually performed in the abdominal area, which has to be cut into small pieces for decellularization. On the other hand, liposuction-derived samples are gel-like tissues that require homogenization and centrifugation as the initial step for separation of the lipid phase. After initial processing, decellularization can be achieved using detergent-based or detergent-free protocols [89].

A detergent-free method for adipose tissue decellularization was described by Flynn et al. in 2010, in which the dECM was produced with a combination of multiple physical and chemical strategies, such as freeze–thaw cycles in hypotonic buffer to loosen the ECM, isopropanol to remove lipids, and enzymatic digestion with trypsin–EDTA, DNase, RNase, and lipase to remove cells and lipids. The resulting dECM conserves the collagen architecture and provides a microenvironment for the differentiation of human adipose stem cells [90,91]. A further published similar protocol demonstrated, by immunohistochemical staining, that laminin and collagen type IV remain abundant in the decellularized matrix. In vitro and in vivo models with microporous foams and hydrogel scaffolds with cells both demonstrate strong support for adipogenesis and induce an angiogenic response and formation of new adipose tissue [55,92]. dECM scaffolds generated by similar detergent-free methods, with a combination of isopropanol, trypsin, EDTA, and DNase–RNAse in gel-like liposuction-derived samples, served as support for human adipose-derived stem cells and adipose regeneration [93].

In adipose tissue, the use of detergents for decellularization seems to increase the risk of matrix protein denaturalization and degradation. Wang et al. reported a method with multiple sequential physical and chemical steps, including a polar solvent extraction and Triton X-100 treatment, which resulted in the maintenance of collagens but the absence of laminin in the final dECM [94]. Similar results were obtained when SDS was used during the decellularization process [95]. Note that although laminin was absent in Triton-X-100-treated samples, in vivo studies have confirmed that the dECM undergoes vascularization and adipose tissue regeneration at Day 30 of implantation, which is consistent with other reports on the adipose tissue-derived matrix.

### 4.4. Skeletal Muscle and Tendons

Muscles are connective tissues formed by contractile fibers. Skeletal muscles are responsible for voluntary movement and homeostasis, and they are attached to bones by collagen fibrillar structures called tendons. Skeletal muscle is divided into several innervated and vascularized subtypes. Given the complex structure of skeletal muscle, it is difficult to pinpoint the exact distribution and composition of the ECM. Collagen is the most common component, as it contains collagen types I, II, III, IV, V, VI, XI, XII, XIV, XV, and XVIII. GAGs are ubiquitous in the ECM, while interactions between proteins and glycans are particularly important to regulate protein distribution. Moreover, ECM glycoproteins and cell membrane–protein interactions transmit the mechanical force in the muscle and are active during muscle injury regeneration [96].

Skeletal muscle loss is often the result of a traumatic injury. In this sense, reconstruction surgery may be required to recover functionality [97]. The first option is always an autologous transfer from nearby tissue, but this implicates a partial loss of functionality or volume. Among other approaches, the use of the muscle’s decellularized ECM is a promising treatment, due to several reasons. Decellularized muscle xenografts are feasible, as a muscle’s ECM is similar among different species, thereby minimizing the risk of the immune response [98,99]. Moreover, the dECM from skeletal muscle shows good integration in vivo, promoting vascularization, remodulation, and differentiation.

The initial skeletal muscle decellularization protocols included physical methods such as freeze–thawing or proteases, which have been further proven too aggressive for proper muscle tissue decellularization. At present, and aiming to preserve the matrix content and tissue structure, some less aggressive detergents, salt solutions, and nucleus-specific enzymes are the preferred decellularization methods. Most of the protocols use weak acids or detergents, such as sodium deoxycholate or Triton X-100 and SDS, respectively, followed by DNase treatment—all of them at low concentrations and exposition times and with multiple repeated cycles [100,101]. In some cases, trypsin is used in low concentrations and for short times, ensuring it does not damage in excess the protein structure.

Tendon tissue is a highly fibroelastic structure that connects muscles to bones. A tendon’s ECM is mainly formed of collagen type I, elastin, and proteoglycans, and it provides mechanical and elastic capabilities. Collagen, in particular, constitutes up to 80% of the dry mass of tendons. These proteins are organized by creating fibers, fascicles, and, finally, the tendon itself. Other than that, there is a huge network of proteoglycans and other elastic macromolecules [49].

Tendons have a natural healing capacity, but they can be damaged if the injury goes beyond this healing capacity. Damage to tendons can be the result of severe trauma or the result of continuously repeated injuries during recovery processes [47]. When using a material to repair possible damage, the main properties required are mechanical and elastic capacities. In this context, dECM derivate materials show good regenerative properties, particularly the ones created with decellularized tendons, as they are assimilated easily and promote new tissue formation.

In order to decellularize tendons, detergents are the most commonly used reagents. Triton X-100 and SDS have been tested and compared independently as tendon decellularization methods, with and without a previous freeze–thawing cycle [102]. Triton X-100 treatment shows less efficiency in cell lysis, with no significant cell removal, and induces damage to the tendon structure. On the other hand, SDS is more effective as a tendon decellularization agent, with less damage to the ECM components and collagen structure [102]. Tri-n-butyl phosphate (TnBP) detergent has also been used for tendon decellularization purposes, with improved results compared to previous methods. Specifically, TnBP treatment results in a significant decrease in cell density, without disruption of the collagen matrix, even when used in relatively high concentrations [47].

### 4.5. Cardiovascular Tissue

The heart is a muscular organ whose function is mainly the pumping of blood through the circulatory system. The ECM of the heart is quite specific and is composed predominantly of collagens (types I, II, and III), fibrillin, hyaluronan, laminin, fibronectin, and proteoglycans [34,103,104]. Due to its composition, it shows great strength, flexibility, and durability [105].

Cardiovascular diseases are the target of multiple regenerative medicine approaches. Stem cell-related approaches have been tested as promising therapies for myocardial infarction, chronic ischemic myocardial dysfunction, and nonischemic dilated cardiomyopathy, among others. Moreover, regenerative and repair strategies have also been investigated as alternatives to heart transplant procedures. On the other hand, research efforts are directed toward defining regenerative or reparative approaches as a substitute to heart valve replacement procedures, a clinically required procedure in certain patients of valvular heart disease (VHD). In all of these cardiovascular regenerative research contexts, decellularized cardiac tissue is now thoroughly assayed, sometimes as a biocompatible and cytokine-carrying implantable scaffold, and in other cases, as a recellularized carrier of therapeutic cells [106,107]. Indeed, whole tissue decellularization, followed by recellularization, is a strategy that many labs are working on [108].

In comparison to other tissues, which are hard to decellularize, current methods yield decellularized heart tissue that retains a great number of its original properties, including its elasticity. The most frequent method to achieve heart decellularization is the use of specific detergents combined with other decellularizing agents. Detergents such as SDS, sodium deoxycholate, PEG, or Triton X-100 work very well as decellularization agents in valves, tissue pieces, and in a whole heart decellularization context. These detergents are often used together [76], but they have also been individually compared and optimized. For example, for whole porcine heart decellularization, Ferng et al. suggested 3% SDS as the optimal detergent, especially when perfused at a pressure between 90 and 120 mmHg, while discouraging the use of CHAPS or OGP due to their inability to successfully decellularize the tissue. Physical and enzymatic methods are often used before or after the detergent-based decellularization step. For example, osmotic shock before decellularization induces loosening of the ECM, while pretreatment with trypsin–EDTA also improves further decellularization steps [30,109,110]. After the detergent-based decellularization step(s), and in order to achieve more porosity in the scaffold, hearts are often freeze-dried [109,110,111], and sucrose may be added to the freezing media in order to avoid damaging or denaturizing biomolecules in the heart scaffold [111]. Focused on heart valve decellularization, postdetergent treatment, enzymes at low concentrations have been used in order to remove the waste of nucleic acids [112], a treatment that does not affect collagen valves or elastane and is compatible with further cell seeding strategies [30].

Some authors claim the most common detergents used as decellularizing agents for hearts may cause ECM denaturation and further loss of mechanical properties. That is why some physical and enzymatic treatments have been assayed with no detergent step for heart decellularization. For example, Seo et al. based their study on the supercritical fluid technology using the scCO_2_–EtOH cosolvent system to decellularize hearts, showing that the supercritical carbon dioxide method maintains higher GAG and collagen levels in the remaining decellularized scaffolds [113]. For heart valves, in terms of enzymatic methods, an accutase solution, followed by nuclease treatment, has been reported [112], while other proteolytic and collagenolytic enzymes combined with nucleases are also able to effectively remove nearly all nucleic acids [112]. Other methods have been assayed, but seem more controversial in terms of the achieved nucleic acid removal, such as the use of a combination of proteases and chelating agents, e.g., trypsin–ethylenediaminetetraacetic acid [112].

### 4.6. Vascular Tissue

Vascular tissue is related to the transport of nutrients, oxygen, CO_2_, hormones, and blood cells through the body. Essentially, it comprises arteries, arterioles, and veins, while only arteries are the common target for decellularization approaches. Arteries are elastic blood vessels that carry oxygenated blood from the heart to the whole body. The main arteries are composed of three tissue layers, from inside out: intima, media, and adventitia. The tunica intima’s ECM contains mainly laminin and collagen type IV, while the medium layer is principally composed of collagen type III, elastin, glycoproteins, and GAGs. In contrast, the ECM of the outermost tunica consists primarily of collagen type I and elastin, but it also contains proteoglycans such as biglycan and decorin, as well as thrombospondin-2 [114]. Although arteries have a very complex structure, it is very important to maintain the ECM components in the interest of keeping the ECM properties intact, such as elasticity and resistance.

Arterial diseases such as pulmonary arterial hypertension, restenosis, and peripheral arterial diseases are now targeted with experimental and promising stem cell therapies [115,116,117,118]. On the other hand, peripheral and coronary artery bypasses are clinical procedures often based on artery replacement by autologous graft transplantation. The use of natural or synthetic biopolymers as grafting materials is a clinically feasible option [119,120], but decellularized artery grafts are gaining increased research attention as artery bypass grafting materials due to their proper molecular and mechanical properties and their reduced immunogenicity [121,122].

Before the artery decellularization technique starts, there are different steps that are recommended for better results. Some protocols suggest to lyse blood cells by washing the arteries in distilled water while shaking [120], while others include three freeze–thaw cycles with EDTA [50]. As in many other tissues, decellularization is mainly based on the activity of detergents such as SDS, EDTA, SDC, CHAPS, Triton X-100, or DOC [27,50,56,120,123,124], used individually or in combination [50]. Some protocols report trypsin and hypo/hypertonic solutions used together with these detergents [27,50,56], while the use of enzymatic DNA and RNA removal as one of the final steps is also recommended [27,56]. All of these chemical methods report quite good results, both in vitro and in vivo, implying the use of detergents as a valuable approach to vascular decellularization, while improvements related to avoiding immunogenicity and cytotoxicity are required [121].

Recently, arterial decellularization mediated by supercritical and pressurized CO_2_ has been described. It briefly consists of a high-pressure syringe pump that delivers liquid CO_2_ and ethanol or limonene (as cosolvents) through a preheated extraction vessel. Samples are treated with scCO_2_ and endonucleases to remove residual cosolvent and DNA. This approach yields a nearly intact decellularized tissue free of cells, lipids, and nucleic acids, proposing an alternative to traditional decellularization methods. Nevertheless, further in vitro and in vivo analyses need to be completed [125].

### 4.7. Dermal Tissue

The skin’s main function is to protect, but it is also in charge of thermoregulation and perception. Dermal tissue is complex, with different layers, such as the epidermis, dermis, and subdermis, each with different compositions, properties, and functions. The epidermis is the outermost layer, and its main function is protection. Behind it, the dermis softens stress and strain, while also provides a sense of touch, elasticity, and heat. The inner layer is the subdermis and it is in charge of insulation. Dermal tissue, as many tissues, is composed of different collagens (85% of the dermal tissue’s ECM), matricellular proteins, elastin, fibrillin, and also other fiber-forming proteins, such as vitronectin and fibronectin, which are necessary for wound healing. The ECM of dermal tissue is also formed by proteoglycans and GAGs, with functions related to hydration and osmosis balance. Examples of these are hyaluronic acid, decorin, and versican [126].

Dermal conditions such as cutaneous burns and scars require regenerative medicine approaches to restore dermal function. These therapeutic targets comprise stem cell transplants, growth factors, and tissue engineering. When scars, burns, and wounds are not able to heal on their own, they require replacement of the dermal barrier. It is very common to find skin equivalent and reconstruction research in the literature using primary human fibroblasts and keratinocytes, regularly supplemented with a collagen type I matrix or ascorbate [127,128]. In terms of decellularized approaches, acellular dermal matrices (ADMs) are widely used in clinical regenerative medicine approaches because of their biological and structural organization. ADMs can be of both animal and human origin, and they have a lot of different applications, such as the regeneration of skin tissue in burn, wound, and scar reconstructions, among others.

The majority of commercial ADMs are based on patented or proprietary decellularization protocols. Among the experimental research examples, the first step is always the mechanical isolation of the dermal layer, which is obtained by individual or combined chemical, physical, and biological methods supplemented with agitation. Dermal tissue can be incubated in hypotonic buffer for cell lysing [129] before the dermal decellularization step, which is usually carried out with detergents such as Triton X-100, DOC, N-lauroylsarcosinate (NLS), or SDS, and often in combination with trypsin, BSA, EDTA, and/or dispase [25,76,124,129,130,131,132]. Detergents can be also combined with acids and bases for the hydrolytic degradation of residual nucleic acids or even hair, but they can damage the ECM [109]. Some protocols describe the use of a further step in endonucleases treatment to remove residual genetic material [129,132].

It is worth noting that there are some other methods that have been reported that are detergent-free, such as osmotic shock and latrunculin B treatment, but they are not mainstream in the dermal tissue decellularization field [132].

### 4.8. Tissues Related to Respiratory System

Taking oxygen and expelling carbon dioxide is a function of the respiratory system, which is formed by multiple organs. Some of them, such as the trachea, lungs, and even diaphragm, have been tested as raw materials for decellularization purposes [133,134,135].

The trachea is related to essential physiological functions, such as airway protection, phonation, and breathing. It is composed of hyaline cartilage, fibrous tissue, respiratory epithelium, and smooth muscle, with cartilage being the most prominent [134]. Trachea damage requiring replacement surgery can be a result of trauma, neoplastic diseases, or congenital stenosis. Tracheal reconstruction in these cases is still a great challenge, while currently, autologous tissue and cell transplantation, with or without additional grafting material, seems the best solution [136,137]. In this sense, decellularized allograft and xenograft trachea is a material that has been tested in in vivo settings. While hyaline cartilage is part of this tissue, its decellularization protocols are slightly different to the ones described for cartilage, being tissue freeze-drying, followed by detergent treatment, the most common [138]. Some protocols add DNases into the detergent treatment step to ensure the elimination of the nuclear material. Commonly, mild reagents are used with several cycles of repeated treatment. According to the reports, this procedure yields mild disruption of the mucosa layer, with preservation of the majority of the remaining tissue structure [139].

The function of the lungs is related to gas exchange from the environment to the bloodstream. The structure of the bronchi in the lungs is similar to the trachea, while air circulates from the bronchi to the bronchioles on its way to alveolar airspace, where gas exchange occurs. The regenerative capacity of the lungs is low, and a broad spectrum of severe lung diseases, such as obstructive diseases, fibrosis, and sarcoidosis, may require a lung transplantation procedure [140]. In this context, decellularized lung tissue has been tested as an experimental alternative to transplantation. Perfusion of detergents such as SDS and Triton X-100 is a technique useful to decellularize mouse and rat cadaveric lung tissue, thereby preserving the vascular and airway structures of the tissue [141,142,143]. Due to the complexity of lung tissue and the need for instant functionality of used implants, a lung’s dECM requires recellularization with epithelial and endothelial cells via cell infusion and bioreactors for ex vivo generation, maturation, and maintenance of the so-called bioartificial lungs. In vivo implantation of these organs in rats yields anastomosis, but long-term success is still to be achieved [143,144].

### 4.9. Tissues Related to Gastrointestinal Tract

The gastrointestinal tract is a complex microenvironment with different parts and multiple related organs. It is in charge of digesting food to extract energy and nutrients and to absorb them. Damage to the gastrointestinal tract or the related organs can be caused by stress, injury, or diseases that affect one or several tissues of the gastrointestinal tract, for example, trauma, surgeries, neoplasia, cancers, fibrosis, inflammatory bowel disease, esophagectomy, and congenital or acquired defects [145]. The complex anatomy of the gastrointestinal tract makes the use of bioengineered scaffolds a difficult task, requiring the use of multilayered structures and the seed of different types of cells, depending on the tissue anatomy [146]. The esophagus and intestines have been the main target of decellularization in gastrointestinal tissue, along with the related organs such as the liver and pancreas, and decellularization has often been complemented with the seeding of functional cells [147]. Research of the esophagus and pancreas is still in the initial stages of development [148]; therefore, here, we focus on intestine and liver tissue decellularization.

The intestine has a complex cellular and matrix architecture with multiple gradients, and it is therefore difficult to replicate using simple scaffolds. The three-dimensional architecture of the intestine is maintained by the ECM, composed of an intricate network of fibrous structural proteins (proteoglycans and glycoproteins), along with fibronectin, laminin isoforms, collagens, and heparin sulfate proteoglycans (HSPGs). Furthermore, multiple cell phenotypes are present in the intestine, including stem cells, pericryptal myofibroblasts, fibroblasts, endothelial cells, pericytes, immune cells, neural cells, and smooth muscle cells [149]. The intestinal stem cell niche is a well-known dynamic environment located at the base of crypts and embedded within a specific ECM in which the intestinal stem cells (ISCs) reside and control proliferation, differentiation, and tissue homeostasis. That is why multiple research efforts have been conducted to bioengineer the intestinal stem cell niche, including the use of decellularized tissue [150]. For intestine decellularization, a combination of chemical and enzymatic solutions (perfusion of sodium deoxycholate, use of DNase, immersion in a hypotonic solution, etc.) is used to remove cells in this tissue, while maintaining the 3D structure [151,152]. Recent studies have shown that the integrin effector protein focal adhesion kinase (FAK) is essential for intestinal regeneration, and thus the preservation of FAK in decellularized tissue scaffolds is essential for regenerative purposes [153].

Hydrogels derived from decellularized intestine are useful for generating endoderm-derived human organoids, such as gastric, hepatic, pancreatic, and small intestine organoids [154]. Moreover, decellularized intestine scaffolds have been used for regenerative purposes in many other tissues, due to their intrinsic ability to induce site-specific in vivo cellular repopulation and regeneration without the need for an in vitro recellularization step. For example, decellularized intestine has been used for regeneration purposes in vascular [155], cardiac [156], dura mater [157], abdominal wall [158], bladder [159], bowel [160], corneal [161], esophagus [162], tendon [163], ligament, cartilage (meniscus) [164], dermal [165], and bone tissues [166].

In recent decades, several methods have been implemented for liver decellularization, aiming to preserve the liver’s major ECM components such as laminin, elastin, fibronectin, collagen types I and IV, and sulfated glycosaminoglycan (sGAG) [167]. The most promising of such methods is the perfusion decellularization method, in which detergents (SDS and/or Triton X-100), hyperosmotic (NaCl), and enzymatic (DNase) solutions are injected intravascularly [168]. Additionally, liver fragments can be immersed into the detergent solutions under mechanical agitation, and the previously described detergent–enzymatic method can be combined with high G-force oscillation treatment to reduce processing times. In such decellularized livers, the structural properties and the protein composition of the ECM are maintained, while they show good biocompatibility and neovascularization in vivo [168,169].

### 4.10. Nervous Tissue

The nervous system is a complex system of specialized cells that connect the parts of the body and coordinate it using signals. The main cells that transmit said signals are neurons, while they require a network of nonneural supportive cells, i.e., glia cells. In this context, the nervous tissue’s ECM is indeed created by glia cells, which protect, isolate, and feed neurons, thereby allowing synapsis. The main structural components of the ECM are collagen (types II and IV) and laminin. Fibronectin guides axon growth, and acetylcholinesterase helps to regulate neural signals [170].

Damage of the nervous system can be caused by multiple conditions, diseases, and injuries, while symptoms related to tissue damage can be multiple, from mild to severe, including the loss of motor functions [171]. The regeneration of damaged nerve connections is a long-time pursued aim and it has been widely assayed using different approaches. There is consensus in defining the properties of the ideal graft for nerve regeneration, which should be of a flexible, thin, neuro-inductive, conductive, and biocompatible structure, capable of promoting axon proliferation and of guiding its growth toward the reconnection of damaged nervous edges [172,173]. To do this, the structure should be able to be molded by Schwann cells, which are specific glia cells related to the guidance of neural regeneration processes. In this context, the usefulness of dECM-derived nerve scaffolds as implantable grafts has been assessed in in vitro and in vivo animal trials, with some promising results [174]. Some studies have shown the capacity of the dECM to promote axon growth and the regeneration of peripheral nerve connections in rats [69,172,175]. The regeneration process requires weeks, and the subjects are in need of rehabilitation in order to begin proper recovery.

For decellularization of nervous tissue, the most commonly used approach is washing with detergents. Note that in the peripheral nerves, the main DNA source is that of Schwann cells, which protect the axons that carry signals. The detergent that shows the best results in this tissue is usually Triton X-200 [28]. A low concentration and prolonged Triton X-200 treatment has been proven successful in eliminating both the axon and Schwann cells, as well as myelin waste. The elimination causes slight damage to the ECM proteins, but it retains good condition of the structure. This treatment is commonly used in combination with an osmotic cell burst, which breaks the cells, facilitating cell waste removal by Triton X-200. Other detergents, such as SDS or Triton-100, are used without osmotic shock [33,171]. Other than these, nucleases have been used in combination with detergents and osmotic methods to ensure that the DNA is properly removed.

### 4.11. Cornea

The cornea is a transparent, avascular, and highly innervated connective tissue that acts as the primary structural barrier to infections, and is the first lens of the eye optical system. The human cornea is organized in five layers, three of which are cellular (i.e., the epithelium, stroma, and endothelium), and two are considered interphases (i.e., the Bowman membrane and the Descemet membrane). This highly organized structure contributes to the cornea’s transparency and mechanical strength, while disruptions to this pattern disturb said transparency and result in loss of vision [176,177]. The cornea’s ECM is composed of water, inorganic salts, proteoglycans, glycoproteins, and collagens. The stromal lamellar collagen fibrils are heterotypic hybrids of types I and V, with significant amounts of collagen types VI, XII, and XIV. A high concentration of small leucine-rich proteoglycans, including decorin, lumican, and keratocan, decorated with dermatan sulfate and keratan sulfate are present in the lamellae, credited with maintaining the interfibrillar spacing required for transparency, and contributing to the regulation of corneal hydration.

Many corneal disorders require a corneal transplant, while obviously there is limited availability of donor tissue. As an alternative to cadaveric corneas, among others, the dECM from acellular porcine and bovine cornea and decellularized amniotic membrane have been combined with different cell types to form full-thickness corneas with stroma, epithelium, and endothelium layers [178,179,180]. This dECM replicates the structure and functional requirements of the native cornea, with the maintenance of the collagen fibril organization, transparency, biocompatibility, suitable mechanical toughness, and low immunogenicity [181,182].

Detergents such as SDS and Triton X-100 were commonly used in the pioneering cornea decellularization methods. Du et al. used a 24 h SDS (0.5% or 1%) treatment to generate a decellularized porcine cornea matrix, which was opaque and swollen after the decellularization [48]. Transparency was restored after soaking in sterile glycerol for one hour, but implantation in a rabbit model showed stromal edema and worsening of corneal opacity throughout the 28-day observation period. Another comparative study used NaCl, 0.05% SDS, or 1% Triton-X100 to decellularize human corneas, and observed that NaCl did not affect transparency, while Triton-X-treated corneas experiencing tissue clouding; meanwhile, SDS-treated corneas appeared the most cloudy/opaque after decellularization [183,184]. Conversely, SDS treatment was combined with benzonase (nuclease) and protease inhibitors in human corneal sheets; in this case, when the dECM was recellularized and implanted in a rabbit model, the implanted tissue maintained complete transparency for three months [185]. A conceptually similar decellularization approach combines sodium N-lauroyl glutamate (SLG) surfactant with supernuclease (a nuclease homologous to benzonase), and also provides adequate transparency and good biocompatibility without degradation 28 days after transplantation [57].

Benzonase endonuclease is often used as the main decellularizing agent in a detergent-free approach, based on its ability to quickly infiltrate the corneal stroma, combined with its easy removal by repeated washes. This approach minimizes the destruction of the ECM, with minimal loss of optical transparency and proper results in animal transplantation assays [186].

### 4.12. Thymus

The thymus is an innervated organ part of the lymphatic and endocrine systems. The function of the thymus is to allow the development and maturation of the T-cell repertoire, and therefore, it has a main role in the immune response. Specifically, T-cell precursors are generated in the bone marrow and migrate to the thymus to become thymocytes, ultimately maturing immunocompetent T-cells. Endothelial and epithelial cells are the main cellular components of the thymus and, along with thymocytes, contribute to creating specific ECMs and microenvironment. The complex interaction network in the thymus includes cytokines, chemokines, matrix metalloproteases, laminin, collagen type IV, and multiple isoforms of fibronectin and glycoproteins, among others, with specific roles and precisely tuned toward the T-cell development process.

Thymus organ cultures are achieved via serial disaggregation and reaggregation of the tissue, and they are useful for ex vivo study of thymus function and complex cell interactions [187]. The rationale of thymus decellularization is mainly related to modeling thymus development, as well as the generation of potential regenerative or therapeutic approaches for in vivo immune response modulation [188]. To this aim, decellularization should be soft enough to keep intact the key ECM components, and should allow further proper recellularization with thymic epithelial cells and endothelial cells. Specifically, the thymus’s dECM-derived bioengineered structure has to be able to reproduce T-cell differentiation and maturation processes. Freeze–thawing, followed by SDS and Triton X-100 detergent treatments, is a common decellularization technique [189]. Thymic epithelial cell-seeded dECM scaffolds, also called thymic reconstructed organoids, have been implanted in immunocompromised mice, yielding the development of populations of mature T-cells overwise absent in these animals [190].

## 5. The Clinical Outcome and Market of the dECM

Translational research is already a reality for some dECM-derived approaches, including several ongoing clinical trials and products on the market (see Table 2). The most common products are decellularized tissue pieces, serving as implantable materials for tissue formation, with proprietary- or patented-specific decellularization procedures [191,192,193].

Decellularized products on the market are generally issued with the ISO standard for biological medical devices (ISO10993-1, the standard for biological evaluation of medical devices), while recently, a specific standard for the evaluation of decellularized products has become available (ASTM F3354-19, Standard Guide for Evaluating Extracellular Matrix Decellularization Processes) [8]. Characterization includes in vitro and in vivo studies to provide data related to the removal of donor DNA and to the safety of implantable commercial products [194,195].

For some specific tissues, there are multiple decellularized products, competing for the same application market and claiming different properties due to differences in decellularization treatments. Comparative clinical case studies are common, and they provide useful information related to clinical success and outcomes of the different commercial dECMs available for each specific application [196,197,198,199]. In this sense, there is a lack of standardized tissue-specific decellularization methods, which would serve as the standard control for comparative purposes [5,200]. Such standardized controls would be useful not only for the assessment of products already on the market, but also to perform more efficient, comparable, and reliable experimental research studies [201].

## 6. Concluding Remarks

Decellularization is a great technique to generate tissue-specific ECM-derived products with multiple applications, including tissue regeneration in clinical settings. Decellularization can be achieved from many tissues, but it has to be designed in accordance with the properties of the target tissue and the intended approach, aiming to preserve specific ECM components. The literature is extensive, but mostly related to empirical experimental research data. As a consequence, a variety of decellularization protocols have been described for each one of the targeted tissues. Therefore, the challenge remains in defining broadly acceptable standardized decellularization and characterization procedures for each specific tissue that would ease the selection of standard controls and the development of future research, ultimately helping in the transfer of knowledge to clinical settings. At present, tissues’ dECM scaffolds are the core of most clinical products, while research efforts are now strongly moving toward the development of postprocessing-related products, such as bioink-related 3D structures. Therefore, we anticipate rapid growth in the number of tissue-specific dECM postprocessing-related clinical products for the coming years.

## Figures and Tables

**Table 1:** Methods used in decellularization processes.

Methods	Mechanism	Side Effects on the ECM	References
Chemical			
Acid; Base	Solubilizes cytoplasmic components, disrupts nucleic acids	Damages collagen and GAG	[32]
Triton X-100	Breaks lipid–lipid and lipid–protein unions, while leaving the protein interactions untouched	Not recommended for ECM where the lipids and GAG are important components	[9,23,26,44,45]
SDS	Liquefies the internal and external cell membranes	Tends to denaturalize proteins and may induce nuclear and cytoplasmic waste in the remaining matrix	[24,25,26,46,47,48]
Triton X-200	Similar to its X-100 counterpart. Very effective in some tissues	Needs to be combined with a zwitterionic detergent to be effective. Damages the matrix in a similar way that SDS does.	[28]
CHAPS	Properties of ionic and nonionic detergents	Similar damage level compared to Triton X-100	[27]
TBP	Disrupts protein–protein interactions	Variable results, collagen degradation but keeping the mechanical properties	[25,49]
Hypertonic and hypotonic solutions	Osmotic pressure to make the membrane explode	High amount of cell waste in the remaining matrix	[11,33,50]
Enzymes			
EDTA, EGTA	Breaks cell adhesion to matrix. It is usually combined with trypsin	Does not actually kill the cells	[29,30,46,50,51]
Trypsin	Digestion of membrane proteins leading to cell dead	Can damage the proteins in the ECM, in particular laminin and GAG	[29,30,43,52]
Pepsin	It targets peptide bounds	Causes high damage in the ECM proteins if left for too long	[31]
Endonucleases and Exonucleases	Degradation of the nuclear material inside and outside of the nucleus	Further cleaning and enzyme removal is required, as they may promote immune response	[6,29,53,54,55,56,57]
Physical			
Freezing	Crystals created in the freezing process destroy the cell membrane	The overall protein structure of the ECM may be compromised	[24,35,36,37,38,58]
Force	Mechanical pressure can be enough to induce the lysis in some tissues	Limited to tissues with hard structures, as it can greatly damage the ECM structure	[39]
Agitation	Commonly used to facilitate chemical agent infiltration and to induce cell lysis	Aggressive processes like sonication can greatly damage the ECM	[40,41]
Vacuum-assisted decellularization (VAD)	Enables chemical agents to reach the more inner parts of the tissue	It is not a decellularization method but a facilitator	[42]
Hydrostatic pressure	Applies high pressure to the tissue and induces cell lysis	Excessive pressure can damage the structure	[43,59]

**Table 2:** Some examples of commercially available tissue-derived ECM products provided by tissue source.

Tissue Source	Application	Examples of Commercial Products
Bone/cartilage tissues	Grafting material for tissue regeneration and orthopedic surgery	-AlloWedge^®^ Bicortical Allograft Bone (RTI Surgical) -Chondrofix^®^ Osteochondral Allograft (Zimmer Inc.) -BioAdapt^®^ DBM (RTI Surgical)
Adipose tissue	Aesthetic soft tissue reconstruction. Multiple tissues.	-Leneva^®^ Allograft adipose matrix (MTF Biologics) -Adipose allograft matrix (AAM) (Musculoskeletal Transplant Found.)
Muscle and tendons	Graft tissue for pelvic organ prolapse	-Suspend^®^ (Coloplast Corp.)
Cardiovascular tissue: heart valve, Pericardium	Graft for valve replacement and aneurysm reconstruction	-Hancock^®^ II, Mosaic^®^ and Freestyle^®^ (Medtronic Inc.) -Prima^®^ Plus and Perimount^®^ (Edwards Lifesciences LLC) -Epic^®^ and SJM Biocor^®^ (St. Jude Medical Inc.)
Vascular tissue: Descending aorta, carotid artery, mesenteric vein, femoral artery.	Xenografting material for arterial replacement, bypass, aneurysm reconstruction, and path graft	-Artegraft^®^ (Artegraft Inc.) -CryoGraft^®^ and CryoArtery^®^ (CryoLife Inc.) -ProCol^®^ (LeMaitre Vascular Inc.)
Nerve tissue	Surgical repair of peripheral nerve discontinuities.	-Avance nerve allograft (Axogen corporation)
Dermal tissue	Grafting matrix for damaged tissue repair	-Dermacell^®^ AWM (LifeNet Health Inc) -Alloderm^®^ RTM (BioHorizons) -AlloPatch HD^®^ (MTF Biologics)
Gastrointestinal tract: small intestine	Xenograft for cardiac tissue repair	-CorMatrix ECM^®^ (CorMatrix^®^ Cardiovascular Inc.)
Others: amniotic membrane, peritoneum	Grafting matrix for damaged tissue repair	-Biovance^®^ (Celgene Cellular Therapeutics) -Meso BioMatrix^®^ Surgical Mesh (MTF Biologics)

## Abbreviations

ECM	Extracellular matrix
dECM	Decellularized extracellular matrix
3D	Three-dimensional
DNA	Deoxyribonucleic acid
GAG	Glycosaminoglycans
SDS	Sodium dodecyl sulfate
CHAPS	3-[(3-cholamidopropyl)dimethylammonio]-1-propanesulfonate
EDTA	Ethylenediaminetetraacetic acid
VAD	Vacuum-assisted decellularization
HA	Calcium hydroxyapatite
MMP	Matrix metalloprotease
BMPs	Bone morphogenetic proteins
DBM	Demineralized bone matrix
DecBM	Decellularized bone matrix
HCl	Hydrochloric acid
ChABC	Chondroitinase ABC
TnBP	Tri-n-butyl phosphate
VHD	Valvular heart disease
PEG	Polyethylene glycol
OGP	n-Octyl-β-D-glucopyranosid
DOC	Deoxycholic acid
SDC	Sodium deoxycholate
ADMs	Acellular dermal matrices
NLS	N-lauroyl sarcosinate
HSPGs	Heparin sulfate proteoglycans
ISCs	Intestinal stem cells
FAK	Focal adhesion kinase
sGAG	Sulfated glycosaminoglycan
SLG	Sodium N-lauroyl glutamate
ISO	International Organization for Standardization
ASTM	American Society of Testing and Materials
